# Association of a Park-Based Violence Prevention and Mental Health Promotion After-School Program With Youth Arrest Rates

**DOI:** 10.1001/jamanetworkopen.2019.19996

**Published:** 2020-01-29

**Authors:** Emily D’Agostino, Stacy L. Frazier, Eric Hansen, Maria I. Nardi, Sarah E. Messiah

**Affiliations:** 1 Department of Family Medicine and Community Health, Duke University School of Medicine, Durham, North Carolina; 2 Miami-Dade County Department of Parks, Recreation and Open Spaces, Miami, Florida; 3 Clinical Science Program in Child and Adolescent Psychology, Florida International University, Miami; 4 Miller School of Medicine, Department of Pediatrics, University of Miami, Miami, Florida; 5 Center for Pediatric Population Health, University of Texas Health Science Center School of Public Health, Dallas

## Abstract

This cohort study estimates the association of a park-based violence prevention and mental health promotion after-school program with youth arrest rates in Miami-Dade County, Florida.

## Introduction

Violence is the leading cause of death and nonfatal injuries in US youth ages 10 to 24 years^1^ and a primary focus of the Centers for Disease Control and Prevention.^2^ Leveraging existing community resources to reduce youth violence in high-crime, low-resource neighborhoods needs to be rigorously tested.

## Methods

Fit2Lead is a park-based violence prevention and mental health promotion after-school program in Miami-Dade County, Florida, developed through extensive cross-agency collaboration for youth ages 12 to 17 years residing in high-crime, low-resource neighborhoods.^3^ The program has been described in depth elsewhere.^3^ This study was approved by the Sterling Institutional Review Board and followed the Strengthening the Reporting of Observational Studies in Epidemiology (STROBE) reporting guideline. Written informed assent or consent was obtained from study participants and guardians.

This prospective cohort study used difference-in-differences models to estimate the association between program implementation and youth arrest rates. The primary factor evaluated was presence or absence of this program (vs other after-school programs) in areas matched by (1) park serving the zip code and (2) baseline youth arrest rates and sociodemographic characteristics (aggregated) drawn from the American Community Survey. The main outcome was change in arrest rates (all offenses) per year among youth ages 12 to 17 years across matched zip codes for 3 years before and after program implementation (2013-2018). Arrest rates were used as a proxy for youth engagement in violent behaviors, consistent with prior literature.^3,4^ Potential confounding variables included area-level sex, age, race/ethnicity, single-parent households, low-income households, and youth perceptions of park safety (binary variable) representing at and above vs below grand mean–centered zip code–level scores using the Teen Environment Neighborhood measure,^5^ Barriers to Activity in Your Neighborhood subscale.

Descriptive statistics were computed for all park area–level sociodemographic characteristics and youth baseline arrest rates across matched zip codes. Preimplementation parallel trends were examined graphically, and potential for reverse causality was tested using crude and adjusted repeated-measures generalized linear models with random intercepts to ensure program site selection was not driven by preimplementation arrests. Differences in youth arrests by zip code before and after implementation were assessed using crude and adjusted repeated-measures Poisson regression models with random intercepts. Statistical significance was set at 2-tailed *P* < .05, and SAS software version 9.4 (SAS Institute Inc) was used for all analyses.

## Results

The program was offered in areas with a population that was 48% male, 60% Hispanic, and 29% non-Hispanic black. In all, 33% of households were single parent and 33% were low income. The program served a mean (SD) of 501 (37) youths per year. Table 1 shows preimplementation youth arrest rates and sociodemographic characteristics. Analyses of preimplementation arrest rate trends showed constant correlation across zip code comparison groups and no evidence of reverse causality (*b* = 0.02; 95% CI, −0.05 to 0.10; *P* = .23 [crude]; and *b* = 0.24; 95% CI, −0.08 to 0.56; *P* = .14 [adjusted]). Sociodemographic variables were included in adjusted models to account for residual differences across matched zip codes. Adjusted difference-in-differences coefficient estimates (Table 2), represented as incident rate ratios, showed a 19.3% greater reduction in youth arrest rates in zip codes where the program was offered (*b* = −0.21 [95% CI, −0.27 to −0.16]) compared with those where it was not. Findings indicated 252 fewer arrests per 10 000 population aged 12 to 17 years over the 3-year intervention period in zip codes where the program was vs was not offered.

**Table 1.** Preprogram Implementation Summary Statistics for 36 Zip Codes With and Without the After-School Program in Miami-Dade County, Florida, 2013-2015

Characteristic	Mean (SD)		
	Zip Codes With Program (n = 12)	Zip Codes Matched by Sociodemographic Characteristics (n = 12)	Zip Codes Matched by Baseline Crime Rate (n = 12)
Juvenile arrest rate^a^	37.78 (22.28)	28.89 (16.21)	32.42 (18.03)
% Low-income^b^	33.36 (15.24)	33.58 (16.13)	37.65 (11.90)
% Male^b^	48.33 (1.50)	50.02 (3.26)	51.39 (3.38)
% Non-Hispanic black^b^	29.38 (15.20)	29.59 (25.91)	23.88 (26.00)
% Hispanic^b^	59.53 (12.89)	56.48 (21.10)	67.09 (26.33)
% of single-parent households^b^	32.54 (5.88)	31.43 (7.41)	31.23 (5.40)
Perception of Safety Score, median (IQR)^c^	16.19 (14.53-17.81)	14.85 (11.88-16.19)	16.19 (14.85-17.00)
Age, median (IQR), y^b^	37.10 (36.70-39.10)	34.95 (32.55-37.70)	34.95 (32.40-41.35)

Abbreviation: IQR, interquartile range.

^a^ Per 10 000 youths ages 12 to 17 years across all targeted zip codes (total population, 34 046).

^b^ Zip code–level demographic data derived from the American Community Survey (2011-2015).

^c^ Perceptions of Safety Scores derived from the Teen Environment Neighborhood measure,^5^ Barriers to Activity in Your Neighborhood subscale; higher score indicates higher perceived safety.

**Table 2.** Adjusted Difference-in-Differences Poisson Regression Estimates of the Association of Program Implementation With Youth Arrest Rates Within 36 Zip Codes in Miami-Dade County, Florida^a^

Characteristic or Measure	Incidence Rate Ratio (95% CI)^a^^,^^b^
After program implementation	0.84 (0.84-0.85)^c^
Program present	2.05 (1.65-2.56)^c^
Program present × after program implementation	0.81 (0.76-0.85)^c^
% Low-income^d^	1.03 (1.02-1.05)^c^
% Male^d^	1.09 (1.03-1.15)^e^
% Non-Hispanic black^d^	1.03 (1.01-1.05)^e^
% Hispanic^d^	1.02 (1.00-1.04)
% Single-parent households^d^	0.96 (0.93-0.99)
Perceptions of Safety Score^f^	0.71 (0.50-0.99)^g^
Median age^d^	1.00 (0.97-1.03)
Differences in least mean squares	
Program present during postimplementation vs preimplementation^h^	0.68 (0.65-0.72)^c^
Program not present during postimplementation vs preimplementation^h^	0.84 (0.84-0.85)^c^

^a^ Estimates reflect change in juvenile arrests per 10 000 youths ages 12 to 17 years across all targeted zip codes (total population, 34 046).

^b^ Adjusted for area-level sex, age, race/ethnicity, single-parent households, low-income, and youth perceptions of park safety (binary variable) representing at and above vs below the grand mean–centered zip code–level scores using the Teen Environment Neighborhood measure.^5^

^c^ Statistically significant at *P* < .001.

^d^ Zip code–level demographic data derived from the American Community Survey (2011-2015).

^e^ Statistically significant at *P* < .01.

^f^ Perceptions of Safety Scores derived from the Teen Environment Neighborhood measure,^5^ Barriers to Activity in Your Neighborhood subscale; higher score indicates higher perceived safety.

^g^ Statistically significant at *P* < .05.

^h^ Estimates for differences in least squares means.

## Discussion

This prospective cohort study found that adjusted youth arrest rate estimates were lower in areas where a park-based violence prevention and mental health promotion after-school program was offered compared with areas hosting other after-school programs. Results suggest that park-based settings can foster positive mental health among youth confronting adversities common to living in high-crime, low-resource neighborhoods^6^ and support growing evidence that leveraging community-based settings through cross-agency collaboration promotes population-level health and resilience.^1,3,6^ Study limitations include residual differences across matched zip codes, although changes in arrest rates (vs counts) were tested, and models were adjusted for multiple area-level factors to control for group differences. In addition, we did not account for spillover effects and concurrent violence-prevention programs. Parks are abundant in many high-crime settings in the United States. Future analyses could allow continued monitoring of outcomes associated with the program to inform potential for scalability in other high-need settings.
